# Region-specific functions of S100A9-derived peptides in inflammation and intracellular delivery

**DOI:** 10.1016/j.gendis.2026.102081

**Published:** 2026-02-21

**Authors:** Hong Han, Xuezhen Ma, Weiyu Chen, Zhiyi Lv, Yexin Yang, Dan Yuan, Yang Mei, Junfeng Shi

**Affiliations:** a Hunan Provincial Key Laboratory of Animal Models and Molecular Medicine, State Key Laboratory of Chemo/Bio-Sensing and Chemometrics, School of Biomedical Sciences, Hunan University, Changsha, Hunan 410082, China; b Shenzhen Research Institute, Hunan University, Shenzhen, Guangdong 518000, China

S100A9, also known as calgranulin B or MRP-14, is a damage-associated molecular pattern (DAMP) protein belonging to the S100 calcium-binding family. It functions primarily as an inflammatory mediator and is abundantly released under pathological conditions, where it contributes significantly to the propagation and maintenance of inflammatory responses.^1^ Notably, beyond its well-established role as a soluble inflammatory mediator, S100A9 exhibits intrinsic cell-penetrating properties that facilitate its shuttling between extracellular and intracellular compartments, including the cytoskeleton, membrane, and nucleus.^2^ This ability is shared by several S100 family members and is thought to be critical for their intra-nuclear functions as transcriptional regulators, thereby initiating inflammatory cascades and amplifying immune activation.^3^ Inspired by this dynamic behavior, we sought to identify the precise structural determinants within S100A9 that mediate its cell-penetrating ability and govern its regulation of inflammation.

Structurally, human S100A9 is composed of four α-helices (H1–H4), which form two EF-hand motifs (helix-loop-helix structures) that mediate calcium binding—connected by a flexible hinge region (Fig. 1A). The N-terminal EF-hand comprises helix H1 (Gln7-Ser23), loop 1 (Val24-Asn33), and helix H2 (Gln34-Asp44), followed by a hinge (Leu45-Asn55) that bridges to the C-terminal EF-hand, which includes helix H3 (Glu56-Leu66), loop 2 (Asp67-Ser75), and helix H4 (Phe76-Arg85). Distinct from other S100 proteins, S100A9 features an extended C-terminal tail (Thr87-Pro114) believed to mediate interactions beyond the S100A8/9 complex, including Ca^2+^-dependent conformational switching, regulation of subcellular localization, and neutrophil immobilization.^4^ Despite these well-mapped domains, it remains unclear which regions enable membrane crossing and inflammation modulation.

**Figure 1:**
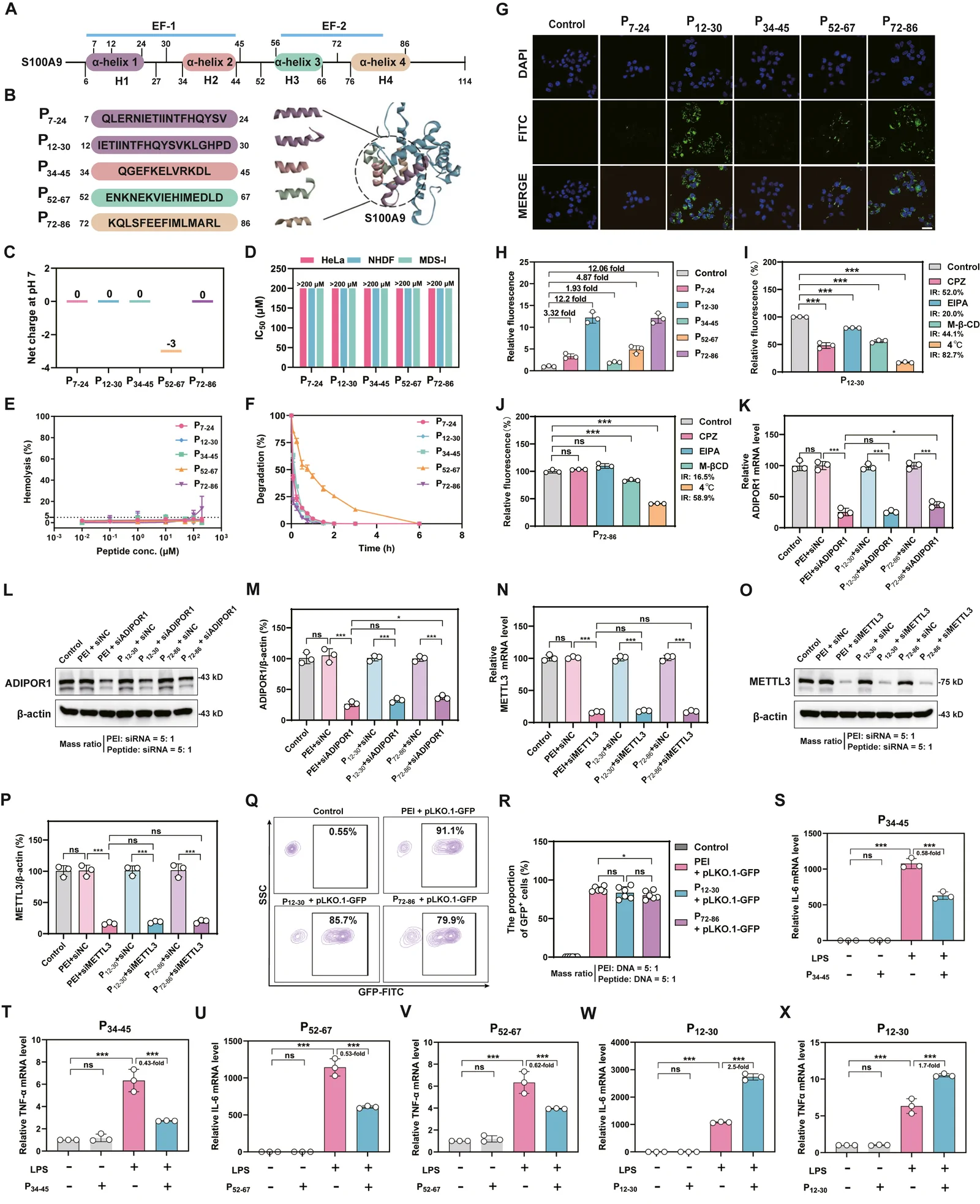
Region-specific functions of S100A9-derived peptides in inflammation and intracellular delivery. **(A)** Schematic representation of the primary structure of human S100A9, highlighting the four α-helices (H1–H4), EF-hand motifs, and the extended C-terminal tail. **(B)** Sequences of synthetic peptides derived from helices H1, H2, H3, and H4 of S100A9, designed to probe the structural contributions of each region to cellular entry and immunomodulatory function. **(C)** Net charge of peptides P_7__–__24_, P_12__–__30_, P_34__–__45_, P_52__–__67_, and P_72__–__86_ at pH 7.0. (**D**) Cell viability assays after 24 h treatment with escalating concentrations of peptides in HeLa, NHDF, and MDS-L cells (*n* = 3). (**E**) Hemolysis assays using primary human erythrocytes to assess the hemolytic potential of each peptide (*n* = 3). (**F**) Proteolytic stability of peptides following incubation with proteinase K (0.03 U/mL) over time (*n* = 3). (**G**) Representative confocal images of HeLa cells treated with 10 μM FITC labeled peptides for 3 h, showing differences in cellular uptake. (**H**) Quantification of peptide internalization by flow cytometry (*n* = 3). (**I**, **J**) Analysis of the uptake mechanisms of P_12__–__30_ and P_72__–__86_ using flow cytometry following treatment with endocytosis inhibitors or incubation at 4 °C (*n* = 3). IR: inhibition rate. **(K)** The mRNA levels of *ADIPOR1* were detected by RT-PCR analysis in HeLa cells (*n* = 3). **(L**, **M)** Western blot analysis of ADIPOR1 expression in HeLa cells transfected with siRNA (si-*ADIPOR1*) (*n* = 3). **(N)** The mRNA levels of *METTL3* were detected by RT-PCR analysis in HeLa cells (*n* = 3). **(O**, **P)** Western blot analysis of METTL3 expression in HeLa cells transfected with siRNA (si-*METTL3*) (*n* = 3). **(Q**, **R)** The proportion of GFP^+^ cells was detected by flow cytometry (*n* = 6). (**S–X**) Quantitative RT-PCR analysis of *IL-6* and *TNF-α* expression in LPS-stimulated RAW 264.7 cells treated with peptides (*n* = 3). Error bars represented as mean ± standard deviation (SD); ns: not significant; ∗*p* < 0.05; ∗*p* < 0.01; ∗∗∗*p* < 0.001.

To explore the functional roles of specific helices in S100A9, we designed a series of peptides spanning key structural motifs: P_7__–__24_ (H1), P_12__–__30_ (H1), P_34__–__45_ (H2), P_52__–__67_ (H3), and P_72__–__86_ (H4) (Fig. 1B). These peptides were synthesized, and their identities and purities were confirmed by analytical HPLC and LC-MS (Fig. S1). Theoretical net charges of the peptides at physiological pH (pH 7.0) were calculated based on their amino acid sequences. Except for P_52__–__67_, which is positively charged, all other peptides were electrically neutral (Fig. 1C). Next, we investigated their biocompatibility and stability. To evaluate cytocompatibility, MTT assays were conducted on HeLa, NHDF, and MDS-L cell lines. All five peptides exhibited IC_50_ values above 200 μM (Fig. 1D), indicating low cytotoxicity and favorable safety profiles. Hemolysis assays further supported their biocompatibility; negligible hemolytic activity was observed for all peptides except P_72__–__86_, which showed modest hemolytic potential (Fig. 1E). Peptide stability was assessed by incubating the peptides (500 μM) with proteinase K, a broad-spectrum endopeptidase. As shown in Figure 1F, all peptides degraded over 120 min, with P_52__–__67_ displaying resistance to proteolytic degradation, retaining approximately 25.4% of its initial concentration. Despite differing proteolytic stability profiles, the S100A9-derived peptides exhibit consistently favorable biosafety and robust physicochemical properties, underscoring their promise for further functional and therapeutic exploration.

Efficient cellular entry or surface binding is a prerequisite for the biological activity of peptide-based therapeutics. To assess the cellular interaction profiles of S100A9-derived peptides, we synthesized fluorescein isothiocyanate (FITC)-labeled versions and monitored their localization using confocal microscopy and flow cytometry (Fig. S2). After 3 h of incubation with 10 μM FITC-labeled peptides, both imaging modalities clearly demonstrated significant intracellular uptake of P_12__–__30_ and P_72__–__86_, whereas P_7__–__24,_ P_34__–__45_, or P_52__–__67_ exhibited minimal internalization (Fig. 1G and H). Interestingly, while most cell-penetrating peptides (CPPs) are cationic (∼83%), both P_12__–__30_ and P_72__–__86_ are electrically neutral. Their atypical physicochemical properties make them prototypes of the underexplored class of neutral CPPs and promising candidates for further development.^5^ Notably, we found that P_52__–__67_ was found to binds to the cell membrane without efficient internalization (Fig. S3). Such distinct subcellular distribution may points to a divergent function in modulating inflammation from P_12__–__30_ and P_72__–__86_.

To elucidate the uptake mechanisms of P_12__–__30_ and P_72__–__86_, flow cytometric analysis was performed under various inhibitory conditions. Incubation 4 °C led to near-complete inhibition of uptake for both peptides, indicating that the internalization primarily occurs through an energy-dependent pathway (Fig. 1I and J). To further probe the endocytic pathways involved, HeLa cells were pretreated with chlorpromazine (CPZ; a clathrin-mediated endocytosis inhibitor), 5-(N-ethyl-N-isopropyl) amiloride (EIPA; a macropinocytosis inhibitor), or methyl-β-cyclodextrin (M-β-CD; a caveolae/lipid raft-disrupting agent), each at an effective concentration for 30 min. CPZ and M-β-CD each reduced P_12__–__30_ uptake by 52.0% and 44.1%, while EIPA had a milder effect (20.0%). In contrast, the internalization of P_72__–__86_ was primarily inhibited by M-β-CD (16.5% reduction), with CPZ and EIPA showing negligible effects. These differential sensitivities were corroborated by confocal imaging (Fig. S4), suggesting that P_12__–__30_ enters via both clathrin- and lipid raft-mediated endocytosis, whereas P_72__–__86_ predominantly utilizes caveolae/lipid raft pathways. These distinct entry routes may contribute to their divergent biological functions.

To evaluate the cargo delivery potential of P_12__–__30_ and P_72__–__86_, we assessed their ability to transport various nucleic acid payloads into cells, including small interfering RNA (siRNA), plasmid DNA, and single-stranded DNA (ssDNA). For siRNA delivery, HeLa cells were treated in opti-MEM medium with P_12__–__30_, P_72__–__86_, or polyethyleneimine (PEI, a widely used commercial transfection reagent), each combined with 1 μg/μL of ADIPOR1-siRNA, METTL3-siRNA, or NC-siRNA. After 12 h, the medium was replaced with complete DMEM, and cells were cultured for an additional 48 h. Transfection efficiency was evaluated by qRT-PCR and Western blot analysis. The results indicated that both P_12__–__30_ and P_72__–__86_ effectively delivered siRNA and reduced target gene expression, with P_12__–__30_ performing comparably to PEI, whereas P_72__–__86_ showed moderately but significantly lower delivery efficiency (Fig. 1K–P). In parallel, plasmid DNA transfection efficiency was analyzed using pLKO.1-GFP and pcDH-GFP constructs delivered via P_12__–__30_, P_72__–__86_, or PEI, as quantified by flow cytometry. Consistent with the siRNA results, P_12__–__30_ mediated transfection efficiency similar to that of PEI, while P_72__–__86_ again exhibited slightly reduced performance (Fig. 1Q, R and Fig. S5A and S5B). Additionally, ssDNA delivery was examined by incubating HeLa cells in serum-free medium with a complex of 1 μM FAM-labeled ssDNA (∼15 kDa) and 10 μM P_72__–__86_. Confocal microscopy confirmed robust intracellular accumulation of the labeled ssDNA (Fig. S5C). Collectively, these findings demonstrate that P_12__–__30_ and P_72__–__86_ serve as effective cell-penetrating peptides capable of facilitating intracellular delivery of diverse nucleic acid cargos, underscoring their potential as electrically neutral alternatives for therapeutic delivery applications.

Given the established role of S100A9 in regulating inflammatory responses, we further investigated the immunomodulatory functions of the candidate peptides. The murine macrophage cell line RAW 264.7, a widely used model for studying innate immunity and inflammation, was stimulated with lipopolysaccharide (LPS) in the presence of 50 μM peptide. After 9 h of treatment, total RNA was extracted and the expression levels of key inflammatory cytokines, *TNF-α* and *IL-6*, were quantified by qPCR. As shown in Figure 1S–X, peptides P_34__–__45_ and P_52__–__67_ significantly suppressed the expression levels of both *TNF-α* and *IL-6*, indicating their anti-inflammatory potential. On the contrary, P_12__–__30_ markedly upregulated the expression levels of *TNF-α* and *IL-6*, suggesting a pro-inflammation effect. Peptides P_7__–__24_ and P_72__–__86_ showed no significant impact on cytokine expression (Fig. S6). These findings are consistent with the dual role of S100A9 in inflammation, highlighting the functional diversity encoded within its distinct structural regions.

This study elucidates the region-specific biological functions and therapeutic potential of peptides derived from key structural domains (H1–H4) of S100A9, building on the protein's intrinsic immunomodulatory and membrane-interacting properties. Notably, P_12__–__30_ and P_72__–__86_ exhibit efficient membrane penetration and facilitate the intracellular delivery of biomolecule cargo, offering biocompatible and electrically neutral alternatives to conventional synthetic transfection reagents. In addition, P_12__–__30_'s ability to enhance the expression of inflammatory cytokines suggests its potential use as an immunostimulatory adjuvant for cancer or infection-related therapies. In contrast, P_34__–__45_ and P_52__–__67_ significantly suppress inflammatory cytokine expression, indicating promising anti-inflammatory activity and potential applications in the treatment of autoimmune or chronic inflammatory disorders.

Together, these findings provide new insights into the structural basis of S100A9's multifunctionality and establish a foundation for the rational design of peptide-based therapeutics targeting inflammation and intracellular delivery.

## CRediT authorship contribution statement

**Hong Han:** Writing – review & editing, Writing – original draft, Visualization, Validation, Methodology, Investigation, Formal analysis, Data curation, Conceptualization. **Xuezhen Ma:** Writing – original draft, Visualization, Validation, Methodology, Investigation, Formal analysis, Data curation, Conceptualization. **Weiyu Chen:** Writing – review & editing, Writing – original draft, Validation, Software, Methodology, Investigation, Formal analysis, Data curation. **Zhiyi Lv:** Visualization, Validation, Investigation, Formal analysis, Data curation, Conceptualization. **Yexin Yang:** Writing – original draft, Visualization, Methodology, Investigation. **Dan Yuan:** Writing – review & editing, Writing – original draft, Visualization, Validation, Supervision, Project administration, Funding acquisition. **Yang Mei:** Writing – original draft, Visualization, Supervision, Project administration, Funding acquisition. **Junfeng Shi:** Writing – review & editing, Writing – original draft, Validation, Supervision, Investigation, Funding acquisition, Formal analysis, Data curation, Conceptualization.

## Ethics declaration

This study did not involve any human or animal procedures. All animal experiments were approved by the Committee for Experimental Animals Welfare and Ethics of Hunan University (No. HNU-IACUC-2021-102).

## Funding

This work was partially supported by the 10.13039/501100001809National Natural Science Foundation of China (No. 32401127 to D.Y., 82170160 to Y.M.), the Natural Science Foundation of Hunan (No. 2024JJ5072 to D.Y., 2022JJ20021 to Y.M.), the 10.13039/501100003453Natural Science Foundation of Guangdong (No. 2024A1515011057 and 2025A1515012954 to J.S). the Science and Technology and Development Foundation of Shenzhen (No. JCYJ20230807122008016 to J.S).

## Conflict of interests

The authors declared no competing interests.
