# Supplementary material for: Region-specific functions of S100A9-derived peptides in inflammation and intracellular delivery
Source: Genes Dis. 2026 Feb 21;14(2):102081. doi: 10.1016/j.gendis.2026.102081 (PMC13654453; doi:10.1016/j.gendis.2026.102081)
Supplement: Supplementary file 1 [file mmc1.docx]

**Supplementary information**

**Region-Specific Functions of S100A9-Derived Peptides in Inflammation and Intracellular Delivery**

Hong Han^#,a^, Xuezhen Ma^#,a^, Weiyu Chen^#,a^, Zhiyi Lv^a^, Yexin Yang^a^, Dan Yuan^a^, Yang Mei^a,^*, Junfeng Shi^a,b,^*

^a^Hunan Provincial Key Laboratory of Animal Models and Molecular Medicine, State Key Laboratory of Chemo/Bio-Sensing and Chemometrics, School of Biomedical Sciences, Hunan University, Changsha, Hunan 410082, China.

^b^Shenzhen Research Institute, Hunan University, Shenzhen 518000, Guangdong Province, China

**This PDF file includes:**

Experimental Section

Figure. S1 to S5

Tables. S1

Appendix. Raw images of Western blot data.

References

**Experimental Section**

### Materials

AM resins were obtained from CS Bio (Shanghai, China). Fmoc-protected amino acid and 2-(1H-benzotriazol-1-yl)-1,1,3,3-tetramethyluronium hexafluorophosphate (HCTU) were obtained from GL Biochem (Shanghai, China) and CS Bio (Shanghai) Ltd. N,N-diisopropylethylamine (DIPEA) and trifluoroacetic acid (TFA) were purchased from Energy Chemical (Shanghai, China). DMEM was purchased from Gibco, and fetal bovine serum (FBS) was purchased from Tansoole. Hoechst was obtained from Thermo Fisher. Thiazolyl blue tetrazolium bromide (MTT) was purchased from Energy Chemical (Shanghai, China). FITC was obtained from Baoman Bio (Shanghai, China). Lipopolysaccharide (LPS) was obtained from Solarbio (L8880, Solarbio, China). Interferon-gamma (IFN-γ) was obtained from Novoprotein Scientific Inc (Cat. No.:C746, Novoprotein, Shanghai, China). EIPA, M-β-CD, and CPZ was purchased from MedChemExpress (Monmouth Junction, NJ, USA; Cat# HY-101840, HY-101461, and HY-12708).

### Synthesis of the Peptides

All of the peptides were synthesized via the standard Fmoc solid-phase peptide synthesis (SPPS) using AM resins and N-Fmoc-protected amino acids and coupling by DIPEA and HCTU. Crude peptides were cleaved from AM resins by cleavage cocktail (TFA/thioanisole/H2O = 9.5:0.25:0.25) for 3 h, and then, the resins were filtered to obtain the filtered solution. The solution was added to ice ether to fully precipitate the peptides. The crude product was obtained by using centrifugation at 3000 rpm for 5 min, and then, the crude peptides were dried by a vacuum pump overnight. All of the peptides were purified by semipreparative HPLC and identified by analytical HPLC, MALDI-TOF, and LC-MS.^1^

### Cell Culture and Viability Assay

HeLa and RAW264.7 cells were cultured in a sterile cell culture incubator at 37 °C and 5% CO_2_, using DMEM or RPMI-1640 medium containing 10% FBS (Tansoole) and 1% penicillin-streptomycin (Adamas life). HeLa cells were seeded in sterile 96 well plates with 5 × 10^3^ cells per well and allowed to adhere overnight in a sterile cell culture incubator at 37 °C and 5% CO_2_ atm. A 10 mM stock solution of the peptide was serially diluted in FBS-free medium to achieve the desired concentrations (0.1 to 500 μM). Then, cells were incubated with 100 μL of each dilution per well. After 24 h, 10 μL of (3-(4, 5-dimethylthiazol-2-yl)-2,5-diphenyl-tetrazolium) bromide solution (MTT, 5 mg/mL in PBS) was added to each well and removed after 4 h of incubation. Then, 100 μL of DMSO was added to each well and incubated for 30 min at 37 °C with gentle shaking. The absorbance was measured at 570 nm by using an ultraviolet (UV) plate reader (Spectra Max M2).

### Hemolysis Assay

We collected fresh blood in an anticoagulant tube by cutting the tail in mice and washed three times with hemolysis buffer (10 mM Tris, 150 mM NaCl, pH 7.4) by immediate centrifugation at 3,460 rpm for 10 min at 4 °C. Then, 75 μL (0.25% v/v solution) of red blood cells (RBCs) in hemolysis buffer was mixed with 75 μL of 2 × peptide dissolved in hemolysis buffer in a 96 well plate. Hemolysis buffer or 1% Triton-X100 containing buffer was used as negative and positive controls. After 24 h of gentle shaking, the plate was centrifuged at 4000 rpm for 10 min at 4 °C to pellet intact RBCs. 100 μL of supernatant was transferred to another 96 well plate. The absorbance was measured at 415 nm using a UV plate reader (Spectra Max M2), and the percent hemolysis was calculated using the following equation: ((Absorbance treated RBC – Absorbance untreated RBC)/(Absorbance triton-X100 treated RBC – Absorbance untreated RBC)) × 100%.^2^

### Stability Assay In Vitro

Peptides (300 μM) were dissolved in PBS buffer (pH 7.4) and then treated with proteinase K (0.03 U/mL) at 37 °C. At the desired times, aliquots were taken and quenched with 5.0% aqueous TFA. The samples were then analyzed and quantified by analytical HPLC. Each time point was performed in triplicate.^3^

### Confocal Imaging

Cells were seeded in sterile confocal dishes (Cat: #CDG35-16, Bioland, China) with 2 × 10^5^ cells and allowed to adhere overnight in a sterile cell culture incubator at 37 °C and 5% CO_2_ atmosphere. Then, the culture medium was removed, and FBS-free DMEM containing a certain concentration of FITC-labeled peptides was added and co-incubated for a certain period. After coincubation, the cells were gently washed three times with sterile PBS buffer (Cat: #FL10210, Bioland, China), and then Hoechst 33342 (Cat#BMD0061, Abbkine, Wuhan, China) was added for 10 min for staining nucleus. Then, the cells were washed with sterile PBS buffer three times again and with 1 mL of FBS-free DMEM. Finally, cell imaging tests were conducted on a Zeiss LSM980 Confocal Microscope.

### Mechanism of Cell Internalization

As described previous,^4^ cells were incubated in a confocal dish for 24 h in advance. First, the inhibitors were diluted with FBS-free DMEM at the following concentrations (EIPA: 60 μM, M-β-CD: 4 mM, CPZ: 15 μM). And the diluent was added to the cells for 1 h of pretreatment. Then, the culture medium was removed, and the mixture with inhibitors (the same concentrations as before) and 10 μM FITC-labeled peptide was added to the cells for 1 h. Next, the cells were washed three times with PBS, and Hoechst was added for 10 min for staining nucleus. Finally, washing again, the cells were then imaged using confocal fluorescence microscopy (CLSM).

### Flow Cytometry Analysis

Cells were seeded in sterile 6 well plates with 3 × 10^5^ cells and allowed to adhere overnight in a sterile cell culture incubator at 37 °C and 5% CO_2_ atmosphere. The next day, cells were incubated with FITC-labeled peptides in serum-free DMEM at the desired concentration for 1 hour at 37 °C. Following incubation, the cells were gently washed three times with sterile PBS to remove excess peptides. Then, the cells were washed with PBS three times and digested using 0.25% trypsin for 5-10 min. The activity of trypsin was then stopped by adding an equivalent DMEM with 10% FBS. The cells were collected and centrifuged at 2000 rpm for 3 min. Next, the supernatant was removed, and 1 mL of sterile PBS was added to wash the cells twice. After washing, the cell precipitate was resuspended with PBS and analyzed by flow cytometry.^5^ The inhibition rate was calculated as follows: [1 - (Fluorescence intensity of treatment group / Fluorescence intensity of control group)] × 100%.

### Peptide delivery efficiency detection

For small interference RNA (siRNA) and plasmid DNA delivery:

**Cell Seeding**: Seed cells into a 6-well plate prior to transfection. Proceed with transfection when the cells reach 70-80% confluence.

**Complex Preparation**: Prepare the DNA-PEI complex as follows per well: Dilute 2 μg of target siRNA and plasmid DNA in 100 μL of serum-free medium and mix thoroughly. Then, immediately add 10 μg of PEI, P_12-30_, and P_72-86_ into the siRNA or DNA dilution, and mix gently. Incubate the mixture at room temperature for 10-20 minutes to allow complex formation. The ADIPOR1-siRNA (sc-60123) and METTL3-siRNA (sc-149387) were purchased from Santa Cruz Biotechnology (Germany).

**Transfection**: Add 100 μL of the DNA-PEI complex dropwise into each well of cells. Gently swirl the plate to ensure even distribution. After 8 hours of incubation, replace the medium with complete culture medium and continue to culture the cells for 48 hours.^6^ Then cells are collected for subsequent experiments.

For ssDNA delivery: HeLa cells were incubated in a confocal dish for 24 h. According to a charge ratio of 10:1, 20:1 or 40:1, peptides and FAM-ssDNA were added to free DMEM and mixed for 15 min. After the delivery period, the cells were washed with PBS three times and stained nucleus by Hoechst. Finally, it was washed again, and the cells were then imaged using confocal fluorescence microscopy (CLSM).

### Western Blot

After transfection, gently wash the cells three times with PBS buffer, then add 60 μL of RIPA lysis buffer (5×, Promega) supplemented with Protein Inhibitor PMSF (Beyotime Biotechnology) to lyse the cells and collect them in a 1.5 mL centrifuge tube. After a few minutes on ice, the cells are centrifuged at 4 °C and 12,000 rpm for 10 min. The supernatant was taken, and the BCA Protein Assay Kit (Beyotime Biotechnology) was used for quantitative detection. Then, 12 μL of loading buffer (5×, GenScript) was added to 48 μL of the supernatant and heated at 100 °C for 10 min. Place the prepared 10% SDS-PAGE gel (GenScript) in the electrophoresis tank, and then add the protein marker and samples to the gel well in order. After running at 70 V until the strip is neat, the strip is run at a constant pressure of 120 V to the bottom edge of the gel with the JY600E system (Beijing Junyi-Dongfang Electrophoresis Equipment Co., Ltd.). Take out the gel and activate the poly(vinylidene fluoride) (PVDF) membrane with methanol for 45 s, then place the sponge, two layers of filter paper, gel, and PVDF membrane in a certain order. Transfer the proteins from the gel to the PVDF membrane at 250 mA for 1.5 h using the JUNYI JY600E system (Beijing Junyi-Dongfang Electrophoresis Equipment Co., Ltd.). Cut the membrane with the target protein according to the position indicated by the Marker, then prepare a 5% milk powder solution, place the PVDF membrane into the blocking solution, and gently block it at room temperature for 1 h. Wash the membranes three times with TBST and incubate them with the anti-ADIPOR1 (R381283, Zen-bioscience, China), anti-METTL3 (bsm-62004R, Bioss, USA) or anti-β-actin antibody overnight at 4 °C with gentle shaking. Wash the membrane three times again and incubate the membranes with horseradish peroxidase (HRP)-conjugated goat antirabbit antibody (Proteintech) at room temperature for 1 h. Wash the PVDF membrane with TBST three times. The bands were detected with chemiluminescence detection (PR30009, Proteintech). After the image was saved, Image J was used for quantitative analysis.^7^

### Quantitative Real-Time qPCR (qRT-PCR)

Total RNA was isolated using the RNA-easy Isolation Reagent (R701-01, Vazyme), and RNA concentration was measured by the NanoDrop 2000 microultraviolet spectrophotometer (1011U, Nanodrop). 1 μg aliquot of RNA was used for reverse transcription to synthesize cDNA with HiScript III RT SuperMix for qPCR (+gDNA wiper, R323-01, Vazyme), and cDNAs were amplified with ChamQ Universal SYBR qPCR Master Mix (Q711-02, Vazyme). The relative expression level of *TNFα* and *IL-6* mRNA was normalized to that of the internal control GAPDH by using the 2^-ΔΔCt^ cycle threshold method. Each sample was tested in triplicate at least three times.^8^ The PCR tubes used in this experiment were purchased from Bioland (Cat: #PCR001N-8F, Bioland, China) The primer sequences of these genes are presented in Supporting Table 1.

### Statistical analysis

The data are presented as the mean ± standard deviation (SD). Unpaired Student’s t-test was used to analyze data from two groups for any significant differences, and one-way ANOVA with Bonferroni’s post hoc analysis was used for multiple comparisons when the data involved three or more groups. All statistical analysis was performed by using GraphPad Prism 8.00 (GraphPad Software Inc). A P-value of less than 0.05 was considered statistically significant. (*p < 0.05; **p < 0.01; ***p < 0.001; ns, no significance.).

**Supplementary Figures**

**Supplementary Figure S1.** **Analytic HPLC and mass spectra of** **P_7-24_, P_12-30_, P_34-45_, P_52-67_ and P_72-86_.**

**Supplementary Figure S2.** **Analytic HPLC and mass spectra of P_7-24_ -FITC, P_12-30_ -FITC, P_34-45_ -FITC, P_52-67_-FITC and P_72-86_-FITC.**

**Supplementary Figure S3.** **Assessment of cellular localization of** **P_52-67._** Confocal images of HeLa cells after incubation with **P_52-67_** (10 μM) at 3 hours. Scale bar: 20 μm or 5 μm.

**Supplementary Figure S4. Confocal microscopy images about the mechanism of P_12-30_ and P_72-86_ internalization.** HeLa cells were incubated with endocytosis inhibitors. Scale bar: 20 μm

**Supplementary Figure S5. The cargo delivery capabilities evaluation of P_12-30_ and P_72-86_. (A-B)** The proportion of GFP^+^ cells was detected by flow cytometry (n = 6). **(C)** Cells were stained with Hoechst, and DNA was labeled with FAM. **P_72-86_** concentration was 10 μM; FAM-DNA concentrations were 100 nM, scale bar: 5 μm.

**Supplementary Figure S6. Peptides P_7-24_ and P_72-86_ showed no significant impact on cytokine expression. (A-B)** Quantitative RT-PCR analysis of IL-6 and TNF-α expression in LPS-stimulated RAW246.7 cells treated with peptides. Error bars represented as mean ± standard deviation (SD) ns: not significant; * p < 0.05; * p < 0.01; *** p < 0.001; n = 3.

**Supplementary Table1**: **The primer sequences of these genes.^9^**

**Appendix. Raw images of Western blot data.**

**Reference**

1.	Han H, Li Z, Feng Y, et al. Peptide Degrader-Based Targeting of METTL3/14 Improves Immunotherapy Response in Cutaneous Melanoma. *Angewandte Chemie (International Ed In English).* 2024;63(48):e202407381.

2.	Guo J, Zhang S, Tao Y, et al. Synthesis of Cationic Cyclic Oligo(disulfide)s via Cyclo-Depolymerization: A Redox-Responsive and Potent Antibacterial Reagent. *Journal of the American Chemical Society.* 2025;147(8):6772-6785.

3.	Li Z, Feng Y, Han H, et al. A Stapled Peptide Inhibitor Targeting the Binding Interface of N6-Adenosine-Methyltransferase Subunits METTL3 and METTL14 for Cancer Therapy. *Angewandte Chemie (International Ed In English).* 2024;63(24):e202402611.

4.	Chen W, Li Z, Zhao C, Zha L, Shi J, Yuan D. Enzyme-modulate conformational changes in amphiphile peptide for selectively cell delivery. *Chinese Chemical Letters.* 2024;35(12):109628.

5.	Li Z, Su M, Xie X, et al. mDia formins form hetero-oligomers and cooperatively maintain murine hematopoiesis. *PLoS Genetics.* 2023;19(12):e1011084.

6.	Yan Q, Jiang X, Xie W, et al. Enhanced Intracellular Delivery by Twisted CC-Loop Conformation in Cyclic Cell-Penetrating Peptides. *Biomacromolecules.* 2025;26(9):5633-5644.

7.	Xie X, Zou A, Zhang L, et al. Rbm38 Deficiency Impairs Erythroid Heme Biosynthesis and Induces Porphyria via Reduced Ferrochelatase Expression. *Blood.* 2025.

8.	Li Q, Ma X, Li Z, et al. Non-essential role of S100A9 in hematopoiesis in primary MPN and MDS mouse models. *Cancer Biology & Medicine.* 2025;22(7):806-811.

9.	Mroueh A, Algara-Suarez P, Fakih W, et al. SGLT2 expression in human vasculature and heart correlates with low-grade inflammation and causes eNOS-NO/ROS imbalance. *Cardiovascular Research.* 2025;121(4):643-657.
